# One Hundred and Seventy Consecutive Cases of Railroad Surgery

**Published:** 1890-07

**Authors:** H. McHatton

**Affiliations:** Macon, Ga.


### Southern Medical Record.

A MONTHLY JOURNAL OF MEDICINE AND SURGERY.

VOL. XX. ATLANTA, GA., JULY, 1890. No. 7.

### Original Articles.

### ONE HUNDRED AND SEVENTY CONSECUTIVE CASES OF RAILROAD SURGERY.*

BY J. MCHATTON, M. D., MACON, GA.

I have been interested in the subject of railroad surgery since I commenced the study of medicine, as my preceptor was surgeon of two Eastern roads, and I held the position of assistant, under him, before my graduation.

It is not my intention in this paper to show how this branch of work should be done, but how it can best be done with our present opportunities. The eventual solution here, as elsewhere, will be the establishment of hospitals, the expense to be borne by the employes. At present, on most of our roads, there is no medical department, and, in the majority of instances, the nearest doctor is called in the emergency. No matter how competent he may be, he is not prepared to treat the case properly and immediately, as he has no reason to keep the necessary appliances on hand. Consequently, valuable time is lost, and insufficient treatment often the result. If he is in city his work is scattered over miles of territory, in boarding houses and cabins, each case requiring a nurse, the consequent expense to the company being enormous. We are

*Read before the Georgia Med. Association, Brunswick, Ga., Apr. 17, '90.

[By an oversight the Author's name appears above as "J. McHatton, M. D." It should be "H. McHatton, M. D."]

a necessary evil to railroad corporations. They are compelled to have us for their own protection. By making a regular medical department they are enabled to secure, at a moment's notice, the services of men familiar with this class of work, (and allow me to say that it presents more peculiarities than one not in the habit of doing it would think.) At the same time they secure the maximum of work at the minimum of expense; also being in a position to gain absolute information in regard to the extent of injuries, and consequently saving the company more in one case than the expense of the department would be in years. At present we have nothing but what we can carry with us, and if there is any provision on any of our roads for moving wounded men I am ignorant of it. The vast majority of our work is done in the worst surroundings, with no competent nursing, and in many instances not only no clean vessels, but not even clean water, such a thing as securing the surroundings that are looked upon as essential for an antiseptic operation in a hospital is impossible. Consequently I confine myself to what I can easily carry so as to be able to operate anywhere regardless of surroundings—to a certain extent aseptically, if not antiseptically.

My time is too limited to allow me to go into the details of the methods used in moving injured men, etc.

The following table of 170 consecutive cases with 338 clinical lesions treated by Dr. Williams and myself, will give a fair idea of the type of work.

Cases proving immediately fatal are excluded from this table.

Number of cases, 170.

Contusions of back, 12; contusions of back, followed by hæmaturia, 2; contusions of shoulder, 12; contusions of wrist, 1; contusions of leg, 5; contusions of chest 5; contusions of arm, 6; contusions of hip, 11; contusions of face, 3; contusions of hand, 3; contusions of foot, 4; ulcers following old contusions, 3; crushed fingers, 90; crushed legs, 3; crushed thumbs, 11; crushed toes, 11; crushed arms, 5; crushed foot, 1; crushed hands, 12; lacerated wounds of face, 10; lacerated wounds of stump of leg, 1; lacerated wounds of foot, 1; lacerated wounds of arm, 3; lacerated wounds of scalp, 8; lacerated wounds of leg, 1; lacerated wounds of lung, 1. Sprain of ankle, 14. Burn of face, 1; burn of hand, 1; burn of leg, 1; burn of foot, 1. Fracture of ossinnom, 3; fracture of humerus, 5; fracture of radius, 6; fracture of ulnar, 5; fracture of Acromion process, 1; fracture of Metacarpus, 4; fracture of malar bone, 1; fracture of base of skull, 1; fracture of ribs, 3; fracture of tibia, 3; fracture of clavicle, 3; fracture of fibula, 4; fracture of femur, 3. Penetrating wound of elbow-joint, 1; penetrating wound of foot, 1. Gunshot wound of neck, 1; gunshot wound of thigh, 1. Dislocation of the radius, 1; dislocation of the humerus, 1; dislocation of the astragalus, 1. Amputations required leg, 4; amputations required fingers, 28; amputations required fore-arm, 3; amputations required arm, 5; amputations required middle of foot, 1; amputations required toe, 1; amputations required thumb, 5.

You will see that the table is headed by sixty-four contusions of various parts of the body. A contusion in the ordinary sense, is not much of an injury, but these were all of clinical importance. If a man gets his shoulder caught between bumpers, and gets an injury that does not cause a fracture, or break of the skin, but is followed by serious œdema and ecchymosis with consequent loss of use of the limb for weeks, it is still a contusion. One of the above cases was in a stooping position when a six-inch beam fell, striking him between the shoulders with sufficient violence to make a comminuted fracture of the tibia and fibula and leaving a contusion extending from the seventh cervical vertebra to the coccyx.

My treatment of these cases is the wet pack until inflammatory action subsides, then friction with a mild liniment, and electricity if there is any indication for it.

The next three cases of ulcers dependent on sluffing following contusions were in that condition when they came into my care, and were treated with the ordinary routine of pressure, grafting and iodoform.

The next item on the table is 133 cases under the head of crushed, of which ninety are fingers.

Taking the latter as an example, you will see that I do not include any finger under the head of fractures. Consequently these injuries cover everything from a laceration of the soft parts from pressure to a complete destruction of the member.

A finger may seem of minor importance to a surgeon, but to a railroad hand who makes his living by hard work, and whose life so often depends on the grasp of his hand, it is a most serious matter, and I make it a cardinal rule never to sacrifice the smallest part of a member that there is any possibility of saving, as it is better surgery in my estimation to save one finger than to cut off a dozen. I have several hands in my memory now, that are the monuments of months of hard, and unpleasant work, bad crushes that would, a few years ago, have been amputated immediately. The injuries followed by sub facial pus accumulations, with pockets everywhere, exfoliation of bone, and the unpleasant work accompanying such conditions, they are neither pretty nor perfect, and at first sight rather a poor advertisement of one's surgical skill. Still I look upon these hands as some of my best surgical results, and their owners to a man are of the same opinion. The finger will serve as a good type of the treatment of these cases.

First, we clean the member perfectly with an antiseptic solution of bichloride. I am in the habit of doing this with the stream from a fountain syringe, then if there is any part that is absolutely destroyed, cut it off, put all parts in as near a normal position as possible, and be as free as the case requires with antiseptic sutures, dry thoroughly and dust freely with iodoform. This may not be an antiseptic, but it certainly retards pus formation. Then bandage carefully with antiseptic gauze, making a moderate amount of pressure to retain the parts in position. Follow this up with a light splint if needed, and cover all with a thick layer of absorbent cotton. Followed by a common bandage. Instruct the patient to wet it with a carbolized solution, if it becomes painful. Don't remove dressing until pain, constitutional symptoms or the saturation of the dressing renders it advisable. If the injury is followed by sluffing, a thick poultice for a few hours will often be of material aid, and the iodoform is the best dressing for the subsequent granulations. Oxide of zinc ointment is also useful where there is need of slight stimulation. By following this line I have saved many fingers when the outlook was anything but promising.

Taking up the twenty-six lacerated wounds, I will simply say that where I have no chance of primary union, my treatment is the same as with the finger, but in all small wounds where I have a chance of primary union, I dispense with the outer dressings and paint the part over with iodoform and collodion, incorporating gauze or cotton with it when extra strength is needed. Of course at this late day there is no use discussing the old question of sutures in scalp wounds, as we use as many as we need with absolutely no fear of the result.

Sprains of the ankle as you see, are common, my rule is moderate pressure and cold applications as in contusions, followed by plaster of paris, Martin's bandage, or the silk stocking, according to the severity of the injury.

Burns, we still treat primarily with Carron oil or carbolized vaseline under absorbent lint, and a thick layer of cotton, followed by grafting, if the surface is large, as soon as we get the proper granulations. It is useless to go into the details of all the forty-two fractures in the table.

The three fractures of the os innominata with two more that I have had in private practice have been treated by position and sand bags.

Those of the humerus were treated by right-angle splint. Those of the radius and ulnar by anterior and posterior splints, usually followed by plaster of paris. Those of the clavicle, by Sayer's method.

Tibia and fibula, by sand bags followed by plaster of paris.

Femur, by Buck's extension, followed by plaster.

Ribs, by the adhesive plaster bandage.

You will notice that my primary treatment of fractures is not plaster of paris. Of course in selected cases it can be put on with safety, but ordinarily it cannot, in my estimation. Most fractures are followed by œdema, which lasts a few days. If the plaster is put on before the oedema takes place, it cannot be left on with impunity. If during the existence of oedema, as that subsides, the plaster becomes too loose and doe's not fully cover the indications, and the repeated putting on and taking off of this class of dressing is not pleasant to the patient or the doctor. Of course if the patient is in your reach you can take the chances, but if he is at a distance, be careful. The experience of some of the best members of our profession has been very sad. Plaster is the most valuable splint we have, but if put on as an immediate dressing it must be carefully watched. As an immediate dressing for a fracture of the leg or fore-arm to move a patient with comfort, a pillow makes an excellent splint.

The fracture at the base of the skull was an interesting case: Bob B., standing on the top of a car, evidently facing the rear, was struck on the back of the head by a bridge.

I saw him two hours after the accident. Large hæmatoma at base of the skull on left side, pulse seventy, deep hepatude, almost amounting to coma, pupils normal. Ice bag to head and dose of calomel.

SECOND DAY.—Paralysis of left arm, pulse 50.

THIRD DAY.—Paralysis of right arm, pulse 54, slight mental improvement; recognizes his mother; no knowledge of events up to five years ago—repeat calomel.

FOURTH DAY.—Calomel acted well, sugar in urine, no recollection of immediate past.

TWELFTH DAY.—Very gradual improvement, slight motion of each hand; still no recollection of immediate past; can't sit up, as both gleuteii are very tender to pressure—pulse 60, no sugar in urine.

TWENTY-FIRST DAY.—All movements, except extension of right arm; remembers events up to day previous to accident. Complete recovery in every respect in about thirty days.

I wish my time allowed me to go more fully into the details of this case. The condition of the intellectual faculties was one of extreme interest. There was a large amount of sugar in the urine. At first there was a question of middle minnengeal hemorrhage from contre coup involving motor area of the left arm, but the supervention of paralysis of the other arm was incompatible with that diagnosis.

I also wish to call attention to the sugar in the urine as a valuable diagnostic point. Paralysis of both arms may have existed sooner than it was discovered, as at first there was no aid from mental power.

In this group there were quite a number of compound and comminuted fractures. They were all treated on the above general principles, as to splints, with the addition of fenistrae when needed, for cleanliness or observation.

That brings us to the few dislocations, gunshot and penetrating wounds in this collection of cases, which it is scarce worth while to go into.

We will now take up the consideration of the amputations; forty-six in number, of which twenty-eight are of the finger. The time of amputation is still one of the mooted points. I have lately seen several articles advising amputation during shock. My rule is, to operate as soon as reaction takes place, and all of these amputations, with the exception of a few of the minor ones, were done at that time. Shock in this class of injuries, is, as a rule, more severe than would be expected, which is supposed to be partially due to the mental condition brought on by the surroundiugs at the time of the injury. I have in consultation decided against operating three times. Dr. Williams informs me that he has held the same opinion in two cases. As each of these cases died within six or eight hours, notwithstanding all efforts that could be made to promote reaction, you can easily imagine their condition. The only case of which I know that was lately operated on during shock died on the table, which would certainly have been the result in my three cases. Take a case of amputation of the leg, as a type. Say we arrive and find the patient in a state of shock. The first thing to do is to put him in as comfortable a condition as the surroundings will permit; use external heat and be free with hypodermics of morphia and whisky. He rallies—then comes the question of when to operate and what operation to do. The railroad surgeon can recognize no regular operation, and cannot be governed by points of election. He is compelled to make the best of what he has, and save as much of the limb as possible.

I recall a case in which I had three long triangular strips of integument with their bases pointing upward, covering a crushed tibia and fibula—their vitality seemed good.

By using them I made an operation that certainly has never been laid down in a text book—got union by first intention, and a first-class stump—avoiding the danger of going into, or above the joint. I heard afterward, that the remark had been made that it would have been better, had the operation been done lower. Granted, and better still, if the leg had never been crushed.

Having decided on the operation, we begin by cleansing the parts thoroughly; shaving off surrounding hair and getting everything as near antiseptic as possible. I use soap and water on surrounding parts, followed by ether and a free use of bichloride solution. The operation being done, first comes the question of ligatures: What shall we use, and how? Early teachings are lasting, and it was always my habit to use the long silk ligature, until I saw three successive amputations, where the healing was so rapid that the ligatures could never be gotten away. That led me to believe if the tissues would tolerate ten or fifteen inches of ligature they certainly would tolerate half an inch, and now I look upon well prepared cat gut or silk, cut off short, as equally good.

Next comes up another non-settled question—drainage. Last year, in my report on surgery, before this body, I advocated the early removal of drainage tubes—from twenty-four to thirty-six hours—giving in that article the reasons. I can only add that the more I use this method, the more convinced I become of its value.

Then comes sutures. I usually use silk, one row of deep coaptation sutures and sufficient superficial ones to insure perfect coaptation of the skin.

Next comes the use of plaster. My views on that subject have not changed. I absolutely condemn it. It is not antiseptic. I feel sure that I have seen flaps sluff from its pressure, and then its removal. Not long since I had occasion to perform a secondary amputation of the thigh for an osteo sarcoma, in the case of a most estimable lady. The previous operation having been performed in one of the largest Southern hospitals. It was followed by months of suppuration, dead bone, etc., and the only dread that she had in regard to the second operation was the removal of the plaster.

Then comes the final dressing. Dust all the parts freely with iodoform, and cover with many layers of antiseptic gauze, followed by a thick coating of absorbent cotton, on top of which goes a firmly applied cotton bandage. You see that I make no mention of the impervious rubber dressings; they have not proved beneficial in my hands. By retaining the excretions of the skin and keeping a constant warm moisture, they act as a poultice. With the exception of removal on account of the drainage tubes this dressing stays on until the parts are healed, provided saturation or constitutional symptoms do not prevent.

I had several special cases of unusual interest that I intended to present, but my time is so limited that I will confine myself to the following one:

J. C.—Two hours after accident; profound shock; pulse 140; fracture of os innom injury to right lung from probable fracture of rib.

Second day, 6 a. m.; no better; beginning emphysema over anterior aspect of the right lung; 11 a. m., emphysema extends all over right side, from buttox to scalp, and is beginning to appear on left side. The emphysema in this case gradually extended from the hips to the top of the head, except a space about the size of a silver dollar on the occipital bone, involving all the body, right arm as far as hand, and as far as the elbow. For three weeks his condition was critical; he having a succession of attacks of coughing, dyspnœa, and collapse. Twice he seemed to be in articula mortis; his respiration would go to sixty, and his circulation would be unaccountable. After the third week his improvement was gradual, but constant, perfect recovery eventually taking place.

There is one more point, and that is the medico-legal aspect of the cases. We are compelled to look upon each one as not only a possible but a most probable medico-legal case. The surgeon occupies a peculiar position. Often he is the only witness who can give the past, present and probable future of the case, the company depending entirely on his opinion in the majority of the cases that are settled by arbitration; the public usually looking—most unjustly—upon him as the hireling, and, consequently, the biased witness of a corporation.

Gentlemen, in those cases you are often put in a most uncomfortable position, and you should avail yourselves of every method in your power to place yourselves so that when your evidence is called for, you can be just to your patient, your employer and yourself. I am in the habit of following these rules:

1st. In a case of any importance, or when there can be any question of diagnosis, avail yourself of counsel, even if you have to pay for it.

2d. Never inquire into the details of an accident, farther than it concerns your professional work. This will save you some annoyance on the witness stand.

3d. And most important, keep notes of every case of railroad injury, no matter how trivial it may seem at the time. These cases are usually postponed as long as possible, and no matter how clear a case is while you are attending it, as time passes, if it is not a most unusnal one, you forget all about it.

While many cases are just—in fact so just that most corporations never allow them to go as far as the court room—you will find, also, if interested in the subject, enough cases that are emphatically not just to convince you that the "get" of Ananias and Sapphira still walk the earth in fair numbers. If you have any doubts on the question, read the history of spinal concussion for the past fifteen years.

Some time ago a lawyer asked me if I remembered a certain case that I had treated some years before. Upon telling him that I had absolutely no recollection of the case, he informed me that the following week I would be called to testify in regard to it. The party, at the trial, with the assistance of some friends, made out a beautiful case of total paralysis—while extracts from my notes read as follows: "First day, bladder and bowels acting; has all movements." "Third day, bladder and bowels normal; motion and sensation perfect." "Twenty-fourth day, out regardless of orders; left on call." My principal reason for watching this case so closely was that it was a bad contusion of the lumbar region, in a man of alcoholic habit, suffering at the time from decided secondary syphilitic lesions, and I feared the possibility of nerve trouble dependent on the three causes. Nevertheless, when the case was mentioned on the street I had no recollection of it.

The possession of a fair library of the standard medical-legal works is most desirable for any one taking an interest in this branch of surgery. The results in these 170 cases were as follows:

Two deaths from tetanus, one following crushed toes, and the other an amputated finger—both reported to this body last year. All the rest, to the best of my knowledge, are able to earn a living, excepting several recent ones that are still under treatment.

Please remember that these are my views on railroad surgery as it is in my section of the State, and not as it should be.

